# miR-140-3p inhibits bladder cancer cell proliferation and invasion by targeting FOXQ1

**DOI:** 10.18632/aging.103828

**Published:** 2020-10-24

**Authors:** Yuan Wang, Junwen Chen, Xia Wang, Kefeng Wang

**Affiliations:** 1 Department of General Surgery, Shengjing Hospital of China Medical University, Shenyang 110004, China; 2 Department of Urology, Shengjing Hospital of China Medical University, Shenyang 110004, China

**Keywords:** miR-140-3p, bladder cancer, *FOXQ1*, proliferation, invasion

## Abstract

Upregulation of the *forkhead box protein Q1* (*FOXQ1*) promotes bladder cancer (BCa) cell growth and metastasis. Factors affecting *FOXQ1* expression at the post-transcriptional level have not yet been identified. We performed cell proliferation, cell invasion, and tumorigenesis experiments to characterize the relationship between *FOXQ1* and *miR-140-3p*. We found that *FOXQ1* was significantly upregulated and *miR-140-3p* was significantly downregulated in BCa tissues. We also identified an inverse correlation between *miR-140-3p* and *FOXQ1* expression in BCa tissues. Overexpression of *miR-140-3p* reduced *FOXQ1* expression, suppressing BCa cell proliferation and invasion. A luciferase assay confirmed that *miR-140-3p* bound to the 3’-UTR of *FOXQ1* mRNA and decreased its expression. In addition, we used a mouse xenograft model to demonstrate that *miR-140-3p* suppressed tumor cell growth in vivo. Our findings suggest that *miR-140-3p* suppresses BCa cell proliferation and invasion by directly decreasing *FOXQ1* expression.

## INTRODUCTION

Bladder cancer (BCa) is one of the most prevalent malignant urological cancers worldwide. In 2019, approximately 80,470 patients were diagnosed with BCa in the United States, resulting in approximately 17,670 deaths [1]. BCa can be categorized into two groups, non-muscle-invasive bladder cancer (NMIBC) and muscle-invasive bladder cancer (MIBC). These cancers have 5-year survival rates of 60% and 90%, respectively [2]. The recurrence rate of BCa is high [3]. Approximately 80% of patients will die within 5 years after distant metastases are discovered [4]. Transurethral resection of bladder tumor is considered to be an effective treatment for NMIBC [5], while radical cystectomy and/or systemic chemotherapy provides limited benefits to late stage MIBC [6, 7].

The majority of transcripts in the human genome are derived from non-coding RNA (ncRNA) [8]. MicroRNA (miRNA) is a class of ncRNA that is 19-22 nucleotides long. These transcripts act as endogenous, post-transcriptional regulators of gene expression by binding to the 3’-untranslated region (3’-UTR), 5’-UTR or coding sequence region of the target mRNA. miRNA performs its functions through either inhibition of protein translation or through mRNA degradation. Microarray expression profiles and miRNA bioinformatics reveal aberrant expression in BCa tissues [9]. miRNA can act as either a tumor suppressor or an oncogene in different types of cancer. It has been reported that *miR-140-3p* functions as a tumor suppressor in breast cancer [10], lung cancer [11] and lymphoma [12].

The *forkhead box protein Q1* (*FOXQ1*) gene is a member of the *FOX* gene family, which encode proteins characterized by a conserved 110-amino acid DNA-binding motif called the forkhead or winged helix domain. Recent studies revealed that upregulation of *FOXQ1* was associated with progression of many human tumors, including those found in hepatocellular carcinoma [13], gastric cancer [14], colorectal carcinoma [15], prostate cancer [16], lung cancer [17], laryngeal carcinoma [18], esophageal cancer [19], pancreatic cancer [20], breast cancer [21], and BCa [22].

In our study, we detected the expression of *miR-140-3p* and *FOXQ1* in BCa tissues. We investigated the regulation of *FOXQ1* by *miR-140-3p*, as well as the biological functions of *FOXQ1* targeted by *miR-140-3p* in BCa cells.

## RESULTS

### FOXQ1 promotes BCa cell proliferation and invasion

**Figure 1:**
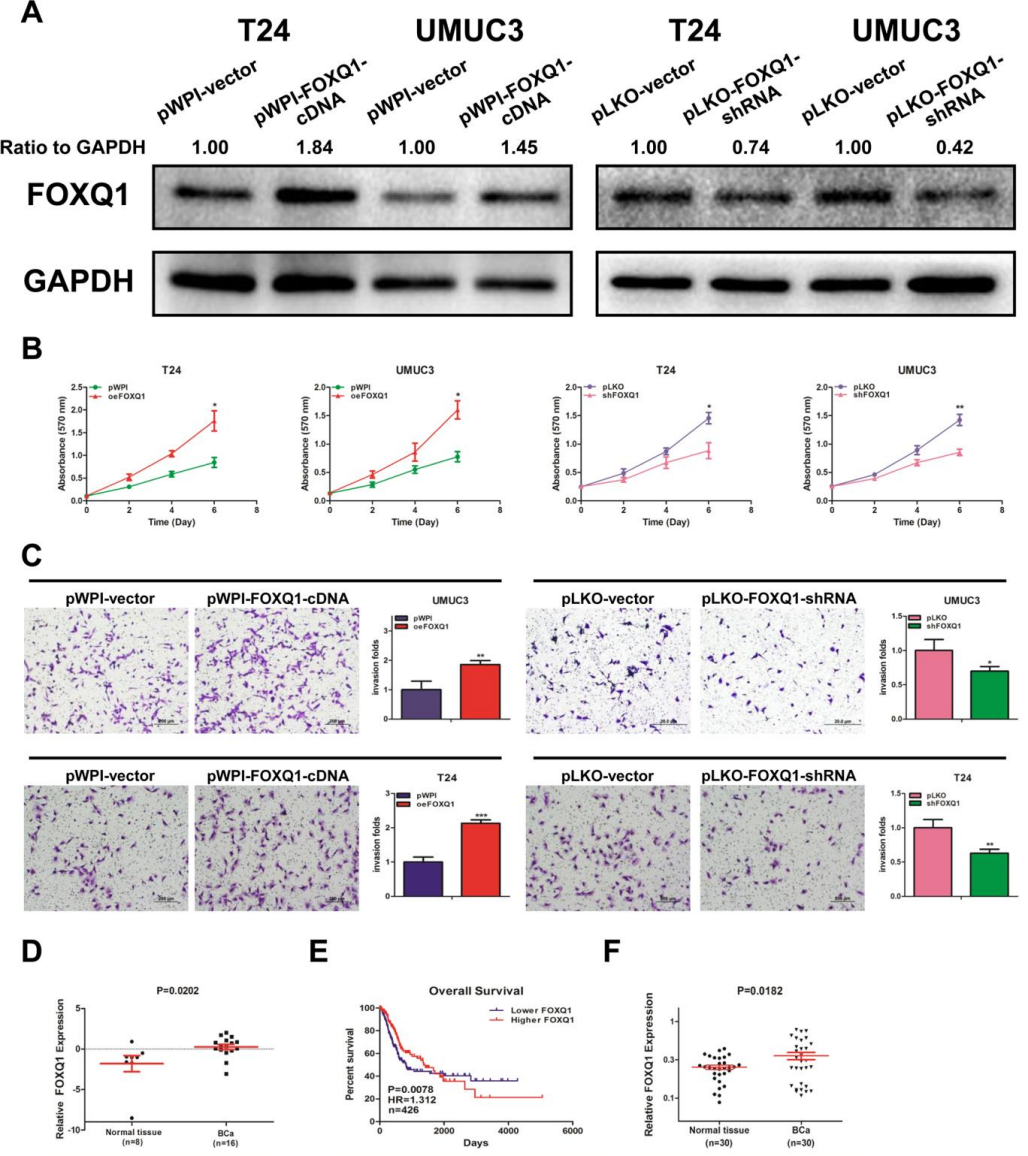
***FOXQ1* promotes BCa cell proliferation and invasion.** (**A**) Verification of *FOXQ1* overexpression and knockdown in T24 and UMUC3 cells by western blot assay. Approximately 50 ug of protein was loaded into each lane 2 to 4 days after transfection. (**B**) T24 and UMUC3 cells were transfected with pWPI-*FOXQ1*-cDNA, pWPI-vector, pLKO-*FOXQ1*-shRNA or pLKO-vector. Cell growth was measured by MTT assay. (**C**) Transwell invasion assays were performed using UMUC3 cells (pWPI and oe*FOXQ1*) and T24 cells (pLKO and sh*FOXQ1*). The invaded cells were counted in 10 randomly chosen microscopic fields (100X) of each experiment and pooled. (**D**) Analysis of microarray sequencing from NCBI GEO Datasets (GSE40355, GPL13497) showed the *FOXQ1* mRNA level in 16 BCa and 8 nonmalignant bladder tissue samples. (**E**) Curves of BCa patient OS were analyzed according to *FOXQ1* expression (data were download from TCGA). (**F**) *FOXQ1* expression in 30 paired human primary BCa tissues and adjacent normal bladder tissues. (**B**–**D**) Each sample was run in triplicate and used in multiple experiments to determine the mean ± SD. *P < 0.05; **P < 0.01 compared to controls.

Knockdown of *FOXQ1* inhibits the migration and invasion of BCa cells [22]. We conducted a western blot assay and confirmed that *FOXQ1* was effectively upregulated or downregulated after transfection of functional *FOXQ1-cDNA* or *FOXQ1-shRNA* in T24 and UMUC3 cells, respectively (Figure 1A). Furthermore, a cell proliferation assay demonstrated that overexpression of *FOXQ1* increased cell proliferation, while knockdown of *FOXQ1* inhibited cell proliferation in T24 and UMUC3 cells (Figure 1B). A transwell invasion assay in T24 and UMUC3 cells revealed similar results (Figure 1C).

Analysis of GEO DataSets (GSE40355, GPL13497) showed that *FOXQ1* expression was significantly higher in 16 BCa tissues than in 8 nonmalignant bladder tissues (Figure 1D). Data from The Cancer Genome Atlas (TCGA) showed that BCa patients with higher *FOXQ1* expression had a shorter overall survival (OS) than patients with lower *FOXQ1* expression (Figure 1E). We then investigated *FOXQ1* expression in BCa samples and adjacent normal bladder samples from 30 patients from our department. The results revealed that the expression of *FOXQ1* was significantly higher in BCa samples compared with adjacent normal bladder samples (Figure 1F). Together, results from Figure 1A–1F indicated that *FOXQ1* expression was higher in BCa samples than in adjacent normal bladder samples.

### miR-140-3p downregulates FOXQ1 expression and suppresses BCa cell proliferation and invasion

miRNA regulates the targeted mRNA at the post-transcriptional level to depress the mRNA degradation or protein translation. Therefore, miRNA might play a vital role in regulating *FOXQ1* expression in BCa. We focused our attention on miRNAs that could decrease *FOXQ1* expression and inhibit BCa cell proliferation and invasion.

**Figure 2:**
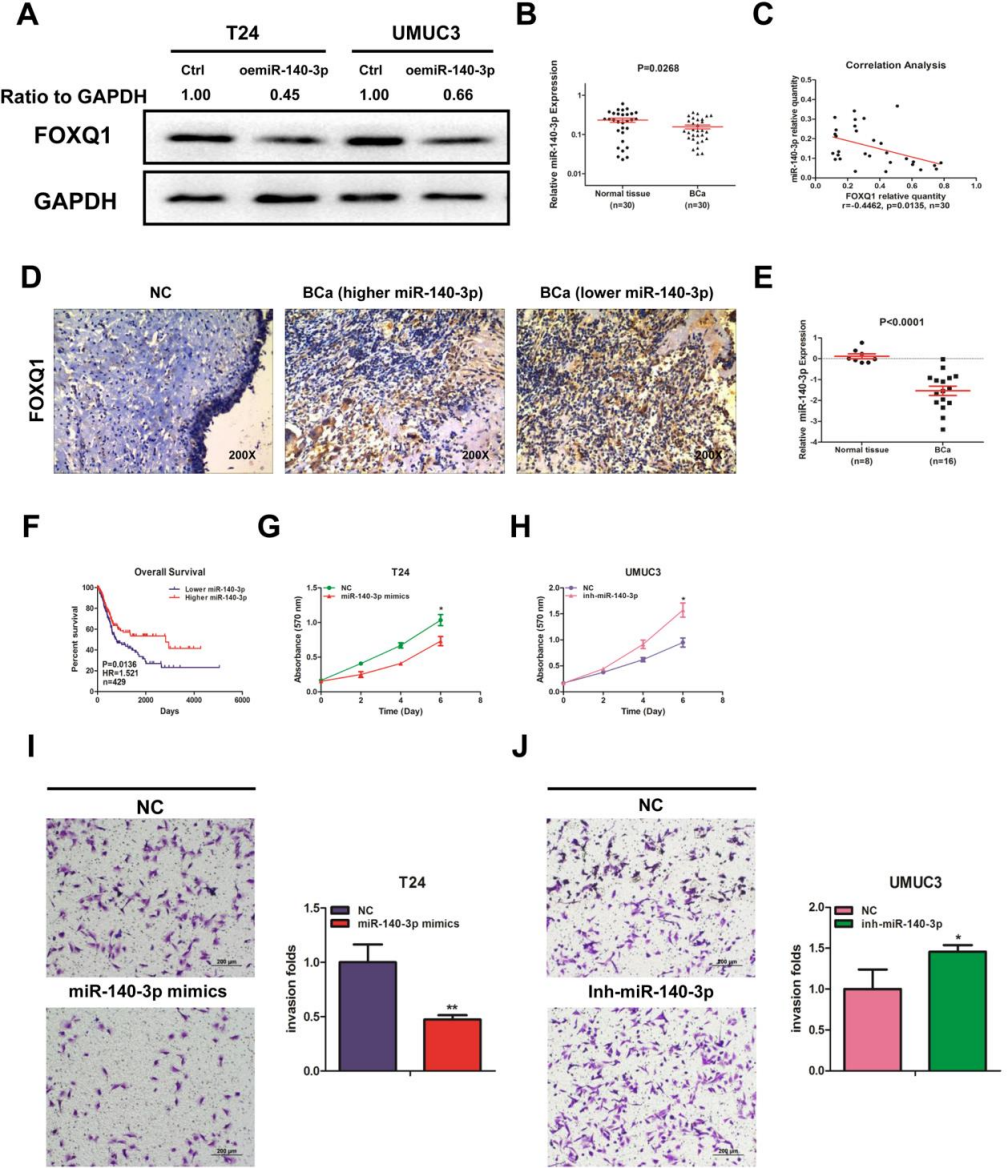
***miR-140-3p* downregulates *FOXQ1* expression and suppresses BCa cell proliferation and invasion.** (**A**) Overexpression of *miR-140-3p* in T24 and UMUC3 cells to determine its effect on *FOXQ1* expression by western blot assay. Approximately 50 ug of protein was loaded into each lane 2 to 4 days after transfection. (**B**) *miR-140-3p* expression in 30 paired human primary BCa and adjacent normal bladder tissues. (**C**) Correlation analysis of *miR-140-3p* and *FOXQ1* mRNA level was performed using the Pearson correlation coefficient. (**D**) Representative immunohistochemical staining of *FOXQ1* in BCa and nonmalignant bladder tissue samples. (**E**) Analysis of microarray sequencing from NCBI GEO Datasets (GSE40355, GPL8227) showed *miR-140-3p* expression in 16 BCa and 8 nonmalignant bladder tissue samples. (**F**) OS curves of BCa patients were analyzed according to *miR-140-3p* expression. Data were download from TCGA. (**G**) T24 cells were transfected with NC and *miR-140-3p* mimics. (**H**) UMUC3 cells were transfected with *miR-140-3p* inhibitor. Cell growth was measured by MTT assay. (**I**, **J**) Transwell invasion assays were performed by transfecting T24 cells with a negative control (NC) or *miR-140-3p* mimics (**I**) and UMUC3 cells with NC or *miR-140-3p* inhibitor (**J**). The invaded cells were counted in 10 randomly chosen microscopic fields (100X) of each experiment and pooled. (**G**–**J**) Each sample was run in triplicate and used in multiple experiments to determine the mean ± SD. *P < 0.05; **P < 0.01 compared to controls.

Using miRNA target-prediction databases (miRDB, Targetscan and MicroCosm), we determined that *miR-140-3p* might target *FOXQ1* mRNA. We explored the effects of *miR-140-3p* on *FOXQ1* expression and found that *miR-140-3p* decreased *FOXQ1* expression in T24 and UMUC3 cells (Figure 2A). We then quantified *miR-140-3p* expression in 30 pairs of BCa samples and adjacent normal bladder samples. The expression of *miR-140-3p* was significantly lower in BCa samples compared with adjacent normal bladder samples (Figure 2B). An inverse correlation was found between the expression of *miR-140-3p* and *FOXQ1* in the 30 paired samples above (Figure 2C). We also observed the expression of *FOXQ1* in patients tissue samples using immunohistochemical staining. The results showed that cells from normal bladder samples expressed lower level of *FOXQ1* than cells from BCa samples. What’s more, we found more positive *FOXQ1* expression in patients with lower *miR-140-3p* expression than in patients with higher *miR-140-3p* expression (Figure 2D). Analysis of GEO DataSets (GSE40355, GPL8227) also verified that the expression of *miR-140-3p* was significantly lower in 16 BCa tissues than in 8 nonmalignant bladder tissues (Figure 2E). We found from TCGA data analysis that patients with lower *miR-140-3p* expression had a shorter OS (HR=1.521, P=0.0136) than patients with higher *miR-140-3p* expression (Figure 2F).

We quantified the expression of *miR-140-3p* in T24 and UMUC3 cells. We found that *miR-140-3p* expression was higher in UMUC3 cells compared with T24 cells (Supplementary Figure 1A). *miR-140-3p* mimics was transfected into T24 cells, while an *miR-140-3p* inhibitor was transfected into UMUC3 cells. We explored whether *miR-140-3p* regulated *FOXQ1* expression in BCa cells. The results of western blot analyses verified that *miR-140-3p* mimics reduced *FOXQ1* expression in T24 cells and the *miR-140-3p* inhibitor increased *FOXQ1* expression in UMUC3 cells (Figure 4A, 4B). qRT-PCR was conducted to verify that *miR-140-3p* was effectively overexpressed in T24 cells and knocked down in UMUC3 cells after transfection (Supplementary Figure 1B). A cell proliferation assay revealed that *miR-140-3p* overexpression significantly decreased the growth rate of T24 cells (Figure 2G). In contrast, *miR-140-3p* knockdown had the opposite effect on the growth rate in UMUC3 cells (Figure 2H). Moreover, we found that the cell invasion activity was significantly inhibited by *miR-140-3p* overexpression in T24 cells (Figure 2I). In contrast, *miR-140-3p* knockdown had the opposite effect on the cell invasion activity in UMUC3 cells (Figure 2J). These data suggested that *miR-140-3p* functioned as a tumor suppressor to inhibit BCa cell proliferation and invasion.

### miR-140-3p directly regulates FOXQ1 expression by targeting the 3'-UTR

**Figure 3:**
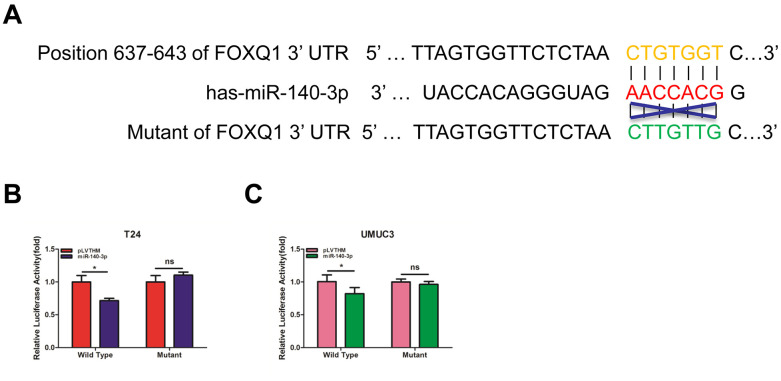
***miR-140-3p* directly regulates *FOXQ1* expression by targeting the 3'-UTR.** (**A**) Sequence alignment of *FOXQ1* 3’-UTR with wild-type (WT) versus mutant potential *miR-140-3p* targeting sites using bioinformatics online databases (miRDB, Targetscan, and MicroCosm). (**B**, **C**) Co-transfection of wild-type or mutant seed regions of *FOXQ1* 3’-UTR constructs with *miR-140-3p* in T24 (**B**) and UMUC3 cells (**C**). The luciferase assay was applied to detect the luciferase activity. *P < 0.05 compared to controls.

To verify that *FOXQ1* is a potential downstream target of *miR-140-3p*, we used online bioinformatics databases (miRDB, Targetscan and MicroCosm) for analysis. The predicted interactions between *miR-140-3p* and the binding sites on the 3’-UTR of *FOXQ1* mRNA are illustrated in Figure 3A. Results from luciferase reporter assays showed that in T24 and UMUC3 cells, transfection of *miR-140-3p* and wild-type sequences of the *FOXQ1* 3’-UTR decreased luminescence intensity. Altering *miR-140-3p* expression did not affect the luminescence intensity of the mutant *FOXQ1* 3’-UTR in either cell type (Figure 3B, 3C). Collectively, these data supported the hypothesis that *miR-140-3p* inhibited *FOXQ1* expression by directly interacting with the 3'-UTR.

### miR-140-3p suppresses proliferation and invasion of BCa cells by reducing FOXQ1 expression

**Figure 4:**
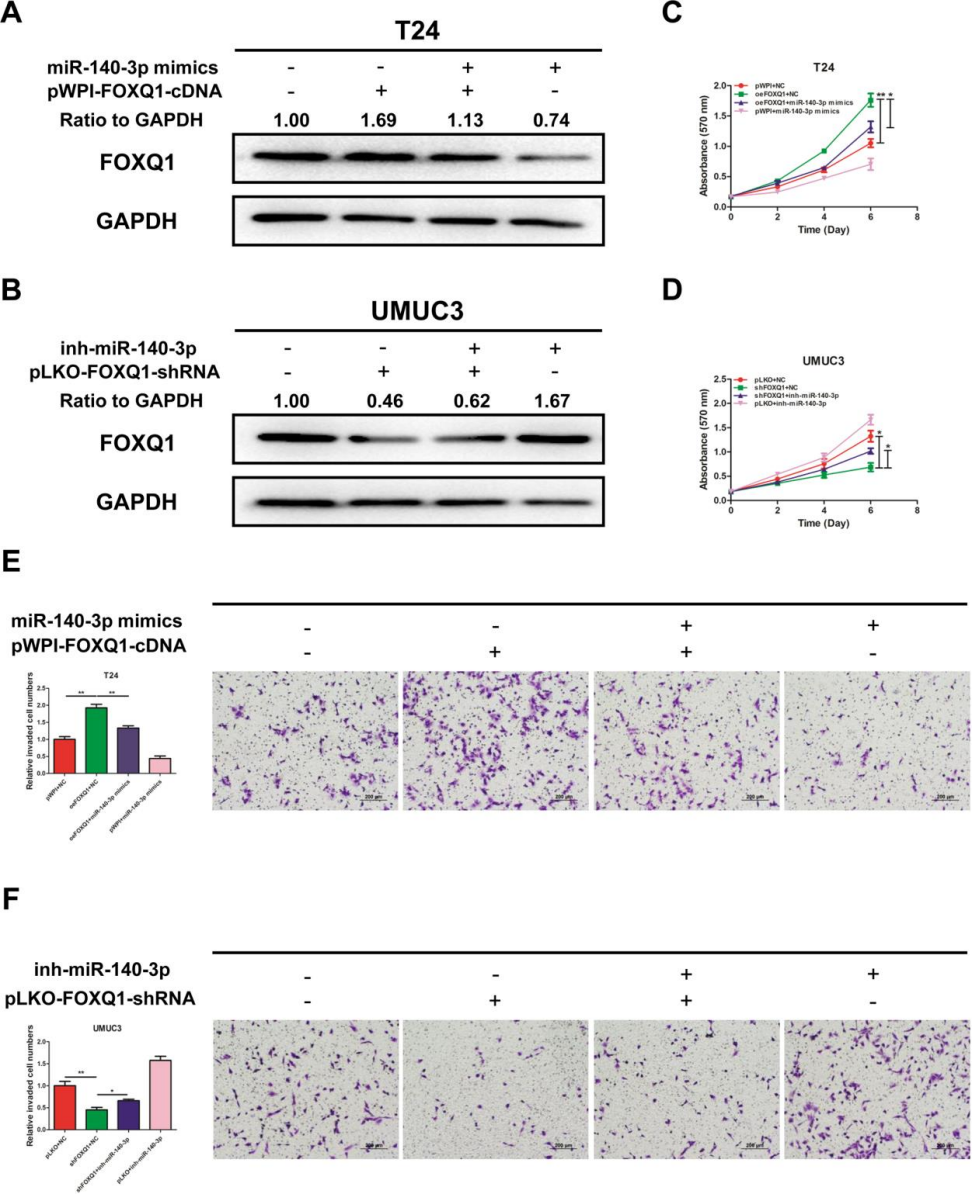
***miR-140-3p* suppresses proliferation and invasion of BCa cells by reducing *FOXQ1* expression.** (**A**, **B**), Western blot assay was performed to detect *FOXQ1* expression. Approximately 50 ug of protein was loaded into each lane 2 to 4 days after transfection. (**A**) T24 cells were transfected with pWPI+NC, *oeFOXQ1*+NC, *oeFOXQ1*+*miR-140-3p* mimics or pWPI+*miR-140-3p* mimics. (**B**) UMUC3 cells were transfected with pLKO+NC, *shFOXQ1*+NC, *shFOXQ1*+inh-*miR-140-3p* or pLKO+ inh-*miR-140-3p* (**C**) An MTT rescue assay revealed that *FOXQ1*-increased cell proliferation could be reversed after adding *miR-140-3p* mimics to T24 cells. (**D**) An MTT rescue assay revealed that sh*FOXQ1*-decreased cell proliferation could be reversed after adding *miR-140-3p* inhibitor to UMUC3 cells. (**E**) A transwell invasion assay revealed that *FOXQ1*-increased cell invasion could be reversed after adding *miR-140-3p* mimics to T24 cells. (**F**) A transwell invasion assay revealed that sh*FOXQ1*-decreased cell invasion could be reversed after adding *miR-140-3p* inhibitor to UMUC3 cells. (**C**–**F**) Each sample was run in triplicate and used in multiple experiments to determine the mean ± SD. *P < 0.05; **P < 0.01 compared to controls.

We explored whether *miR-140-3p* regulated *FOXQ1* expression in BCa cells. The results of western blot analyses verified that *miR-140-3p* mimics reduced *FOXQ1* expression in T24 cells and *miR-140-3p* inhibitor increased *FOXQ1* expression in UMUC3 cells (Figure 4A, 4B). To determine whether *miR-140-3p* is involved in the *FOXQ1*-induced promotion of BCa cell proliferation and invasion, we conducted rescue experiments in T24 and UMUC3 cells. The results showed that *miR-140-3p* mimics partially reversed the increase mediated by overexpression of *FOXQ1* on T24 cell proliferation (Figure 4C). In UMUC3 cells, a *miR-140-3p* inhibitor partially reversed the proliferation suppression mediated by knockdown of *FOXQ1* (Figure 4D). We obtained similar results in both T24 and UMUC3 cells using an invasion assay in place of the cell proliferation assay (Figure 4E, 4F). These data verified that *miR-140-3p* suppressed *FOXQ1*-induced proliferation and invasion of BCa cells.

### miR-140-3p suppresses the growth of BCa cells in vivo

**Figure 5:**
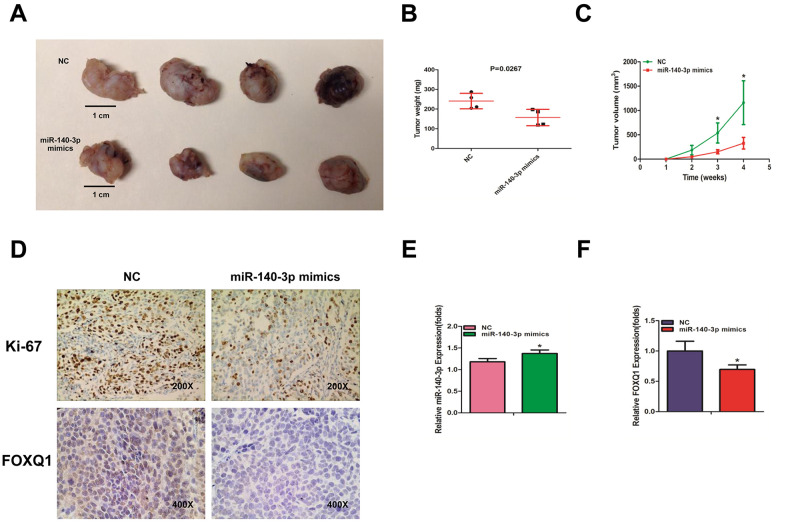
***miR-140-3p* suppresses the growth of BCa cells in vivo*.*** (**A**) Macroscopic appearance of murine tumor xenografts. (**B**) Weights of tumors in 2 groups were measured using electronic scales. (**C**) Summary of tumor volume, which were measured weekly. (**D**) Representative immunohistochemical staining of *Ki-67* and *FOXQ1* in murine BCa cell xenografts. (**E**) The expression of *miR-140-3p* in xenografts was detected using qRT-PCR. (**F**) The expression of *FOXQ1* in xenografts was detected using qRT-PCR. (**B**–**F**) Each sample was run in triplicate and used in multiple experiments to determine the mean ± SD. *P < 0.05; **P < 0.01 compared to controls.

To identify the effects of *miR-140-3p* on BCa cell growth, we transfected T24 cells with either *miR-140-3p* mimics or a negative control. The cells were injected into nude mice subcutaneously. Tumor volume was monitored weekly. The mice were sacrificed 4 weeks after injection. The weights and volumes of the xenografted tumors were measured. As expected, *miR-140-3p* mimics dramatically decreased the tumor weights and tumor volumes compared with those of the negative control group (Figure 5A–5C). Our immunohistochemical staining demonstrated that both *Ki-67* and *FOXQ1* expression was significantly lower in the *miR-140-3p* mimics group than in negative control group (Figure 5D). We performed qRT-PCR to confirm that the expression of *miR-140-3p* increased in the *miR-140-3p* mimics group more than in negative control group (Figure 5E). The result of qRT-PCR also suggested that *FOXQ1* expression was lower in the *miR-140-3p* overexpression group (Figure 5F). Together these data demonstrated that *miR-140-3p* suppressed the growth of BCa cells in vivo and might serve as a therapeutic marker in bladder cancer.

## DISCUSSION

The *forkhead* gene in *Drosophila* and the *hepatocyte nuclear factor 3 alpha* gene in rats were the first *FOX* family genes to be identified [23]. Members of the *FOX* gene family exist in a large range of organisms, functioning in a variety of tissues and playing vital roles in various biological processes. The *FOX* genes are involved in embryonic development [24], cell-cycle regulation [25], tissue-specific gene expression [26], cell signaling [27] and tumorigenesis [28].

The human *FOXQ1* gene was first isolated and characterized in 2001 [29]. The *FOXQ1* gene is expressed strongly in the trachea and stomach, and to a lesser degree in the salivary gland and bladder. In 2010, researchers found that in colorectal cancer, *FOXQ1* promoted tumorigenicity and tumor growth by upregulating p21 expression, which in turn enhanced angiogenesis and anti-apoptosis. It has since been revealed that *FOXQ1* functions as an oncogene in many human cancer types, including BCa [22, 30].

In our study, we found that the expression of *FOXQ1* was higher in BCa tissues than adjacent normal bladder tissues. *FOXQ1* promoted BCa cell proliferation and invasion. We also found that miRNA regulates *FOXQ1* mRNA at a post-transcriptional level to repress mRNA degradation or protein translation. Investigators have discovered that *miR-124*, *miR-506* and *miR-342-3p* suppressed nasopharyngeal carcinoma cell proliferation and metastasis by targeting *FOXQ1* [31–33]. Other researchers have revealed that *miR-1271*, *miR-345* and *miR-519* inhibited *FOXQ1* in gastric cancer [14, 34, 35]. In colorectal cancer, *miR-320*, *miR-342* and *miR-106a* restrained tumor cell growth by targeting *FOXQ1* [36–38]. Finally, lncRNA MALAT1 promoted BCa proliferation and metastasis by targeting *miR-124/FOXQ1* expression [30].

A single gene can be targeted by multiple miRNAs to initiate various functions. We explored whether other miRNAs regulated *FOXQ1* expression to influence BCa proliferation and invasion. We used in silico analysis to identify *miR-140-3p* as a candidate that regulated *FOXQ1* expression in BCa cells. By literature review, we found that *miR-140* had been shown to perform anti-cancer functions in many types of human cancer. Xie et al*.* reported that *miR-140* suppressed *PD-L1* and *cyclin E* expression to inhibit cell proliferation in non-small cell lung cancer (NSCLC) [39]. Fang et al. found that *miR-140-5p* downregulated *YES1* expression to inhibit proliferation, invasion and migration of gastric cancer [40]. Lv et al. revealed that lncRNA-Unigene56159 silenced *miR-140-5p* to de-repress the expression of *Slug*, promoting cell invasion and EMT in hepatocellular carcinoma cells [41]. Wang et al*.* reported that *miR-140-5p* inhibited the proliferation, migration and invasion of BCa cells [42]. Here we found that *miR-140-3p* directly inhibited *FOXQ1* expression in BCa cells. In addition, we determined that *miR-140-3p* inhibited *FOXQ1* expression by targeting the 3'-UTR. Gain- and loss-of-function analysis revealed that *miR-140-3p* suppressed proliferation and invasion of BCa cells by reducing *FOXQ1* expression. Furthermore, *miR-140-3p* inhibited the growth of BCa cells in a xenograft mouse model. These results revealed a network involving *miR-140-3p* and *FOXQ1* that fine tunes the invasion and proliferation of BCa cells.

We found that the expression level of *miR-140-3p* was significantly lower in BCa tissues than in adjacent normal bladder tissues. We found similar results in GEO DataSets (GSE40355, GPL8227). Using TCGA database analysis, we found that patients with higher *miR-140-3p* expression had longer disease-free survival periods than patients with lower *miR-140-3p* expression. *miR-140* was also downregulated in types of cancer [43–45]. The above results suggested that *miR-140* might work as a tumor suppressor miRNA in cancer.

It is well known that one miRNA can target multiple genes. For example, *miR-140* suppresses tumor proliferation and metastasis by targeting insulin-like growth factor 1 receptor in NSCLC [46]. *miR-140* also suppresses cell proliferation and invasion by targeting *ATP8A1* in NSCLC [11]. To explore the role of this newly identified pathway in bladder tumorigenesis signaling, we upregulated *miR-140-3p* expression and found that the growth and invasion of BCa cells were inhibited, which imitated the function of *FOXQ1* reduction by targeted siRNA. Furthermore, the overexpression of *FOXQ1* partially attenuated the anti-proliferative and anti-invasive effects of *miR-140-3p* on BCa cells.

miRNA plays important roles in tumor onset and progression. Recent studies revealed that replacement of tumor suppressive miRNA or inhibition of oncogenic miRNA were possible strategies in cancer therapy [47]. In our study, we found that *miR-140-3p* downregulated *FOXQ1* expression both in vitro and in vivo. Flamini et al. found that *miR-140* replacement treatment combined with other drugs enhanced drug efficacy by reducing the invasion and migration ability of NSCLC [48]. Hence, *miR-140-3p* mimic replacement therapy is a candidate for BCa treatment.

## MATERIALS AND METHODS

### Human tissue specimens

Human BCa tissues and normal bladder tissues were acquired from patients who had undergone surgery and were diagnosed with BCa by pathologists in Shengjing Hospital of China Medical University (Shenyang, China) between 2014 and 2017. Informed consents were signed by each patient before using the tissues. The ethics approval was authorized by the Ethics Committee of Shengjing Hospital of China Medical University.

### Reagents

*FOXQ1* antibody was purchased from Biorbyt Ltd (host: rabbit; catalog number: orb77456 for western blot and orb53843 for IHC). *GAPDH* antibody (0411) was purchased from Santa Cruz Biotechnology (host: mouse; catalog: sc-47724). Anti-mouse/rabbit second antibodies were from Invitrogen (Grand Island, NY). *miR-140-3p* mimic (sequence: UACCACAGGGUAGAACCACGG) and *miR-140-3p* inhibitor (sequence: AGGCGAAGGAUGACAAAGGGAA) were purchased from Biomics Biotechnologies. The antibodies were kept at -20°C.

### Cell culture and transfection

The BCa cell lines (T24 and UMUC3) were purchased from the American Type Culture Collection (ATCC; Manassas, VA) and cultured with DMEM (Invitrogen, Grand Island, NY) containing 10% fetal bovine serum (FBS), penicillin (25 units/ml), 1% L-glutamine, and streptomycin (25 g/ml). The cells were maintained at 37°C in a 5% CO_2_ humidified atmosphere. Cells were detected and identified as mycoplasma and bacteria free for 3 months following the ATCC's instructions.

### Lentivirus packaging

The pWPI; pWPI-*FOXQ1*-cDNA (F-primer: GTGAGGAATTTCGACATTTAAATTTAATGAAGTTGGAGGTGTTCGT, R-primer: TCCTGCAGCCCGTAGTTTTCAGGCTAGGAGCGTCTC); pLKO.1; pLKO.1-*FOXQ1*-shRNA (F-primer: CCGGCGAGTACCTCATGGGCAAGTTCTCGAGAACTTGCCCATGAGGTACTCGTTTTTG, R-primer: AATTCAAAAACGAGACCTCATGGGCAAGTTCTCGAGAACTGCCCATGAGGTACTCG); pMD2G envelope plasmid and psPAX2 packaging plasmid were transfected into HEK 293 cells using the standard calcium chloride transfection method. The lentivirus soups were collected after incubation 48 hours and 72 hours and frozen at -80°C for use.

### RNA isolation and quantitative real-time polymerase chain reaction (qRT-PCR)

Total RNA was extracted from cells and tissues by Trizol reagent (Invitrogen, Grand Island, NY) according to the manufacturer’s protocols [49]. Complementary DNA was synthesized from RNA reverse transcription by Superscript III transcriptase (Invitrogen, Grand Island, NY). qRT-PCR was conducted using a Bio-Rad CFX96 system. GAPDH was used as an internal control. miRNA was isolated with a PureLink miRNA kit (Invitrogen, Grand Island, NY). RPL32 and U6 were used as endogenous controls. All primers were designed as follows: *FOXQ1*: F-primer: CTACTCGTACATCGCGCTCA; R-primer: ACCTTGACGAAGCAGTCGTT; *miR-140-3p*: TACCACAGGGTAGAACCACGG.

### Cell proliferation assay

Transfected BCa cells were plated in 24-well plates (2000 cells/well). At day 1, 2, 3, and 4, 50 μL of 10 mg/mL MTT reagent was added to the wells. After 2 hours of incubation, the medium was sucked out and 500 μL DMSO was added. The absorbance was measured at 570 nm.

### Cell invasion assay

The upper chamber was coated with 100 μL Matrigel (Corning), which was diluted at 1:20 for T24 cells and 1:30 for UMUC3 cells. The chamber was incubated for 2 hours before cells were plated. The transfected BCa cells were suspended in serum-free medium and seeded into the upper chambers of each transwell (8.0 μM pore size) at a 1x10^5^/mL concentration. Then 750 μL medium containing 10% FBS was added to the bottom chambers. The cells were incubated at 37°C with 5% (v/v) CO_2_ for 18 hours (for T24 cells) or 24 hours (for UMUC3 cells). The invaded cells on the lower surface were permeabilized with methanol and stained with 0.1% crystal violet in the dark. The stained cells were photographed and counted under a microscope.

### Western blot assay

Cells were lysed with RIPA lysis buffer on the ice. Proteins were collected and quantified with BCA analysis. Approximately 50 ug protein was loaded into each lane 2 to 6 days after transfection, separated by a 10% SDS/PAGE gel and transferred onto a PVDF membrane (Millipore, Billerica, MA). After incubation with FOXQ1 antibody (dilution, 1:1000) or GAPDH antibody (dilution, 1:1000) overnight at 4°C, the proteins were then incubated with secondary antibodies (dilution, 1:5000) at room temperature for 1 hour. After samples were washed with TBST, the signals were visualized using a chemiluminescent detection system (ThermoFisher Scientific, Rochester, NY) and analyzed with Image Lab software.

### Luciferase reporter assay

Cells were co-transfected with *miR-140-3p* mimics and plasmid containing 3’-UTR sequences of *FOXQ1* wild-type or mutant fragments using the Lipofectamine 3000 reagent (Invitrogen, Carlsbad, CA) according to the manufacturer’s instruction. Dual-luciferase activity was measured 36-48 hours after transfection by a dual-luciferase reporter assay system (Promega, Madison, WI) according to the manufacturer’s instruction.

### Xenografts in nude mice

The animal experiments were performed according to the institutional ethics guidelines approved by the Animal Care Committee of China Medical University. Eight nude mice were purchased from Shanghai Laboratory Animal Center Co. Ltd (China). T24 cells were transfected with *miR-140-3p* mimics or a negative control. T24 cells (2×10^6^) were injected subcutaneously into the posterior flank of the mice (5-6 weeks, 4 mice per group). Tumor sizes were monitored once a week by measuring the length and width with calipers. Tumor volume was calculated by the following formula: volume = (length x width^2^) x 0.52 [50]. Mice were sacrificed 4 weeks after injection, and tumor weights were measured. qRT-PCR was conducted to quantify *miR-140-3p* and *FOXQ1* expression in xenograft tumors.

### Immunohistochemical staining

Each tissue was cut to 5 μm section. The slide was treated for antigen retrieval and incubated with primary antibody. After rinsing with tris-buffered saline, the slide was incubated with secondary antibody, and then incubated with enzyme conjugate horseradish peroxidase. Finally, the slide was counter-stained with hematoxylin.

### Statistical analysis

Statistical analyses were performed using SPSS 17.0 (SPSS, Chicago, IL). Data were expressed as mean ± standard deviation (SD) from at least 3 independent experiments. *P* < 0.05 was considered statistically significant. Data differences were identified by chi-square test or Student’s *t*-test. Pearson correlation analysis was used for comparing data sets. Kaplan–Meier curves were used for survival analysis.

## Supplementary Material

Supplementary Figure 1
