# Supplementary material for: miR-140-3p inhibits bladder cancer cell proliferation and invasion by targeting FOXQ1
Source: Aging (Albany NY). 2020 Oct 24;12(20):20366–79. doi: 10.18632/aging.103828 (PMC7655201; doi:10.18632/aging.103828)
Supplement: Supplementary Figure 1 [file aging-12-103828-s001..pdf]

SUPPLEMENTARY FIGURE

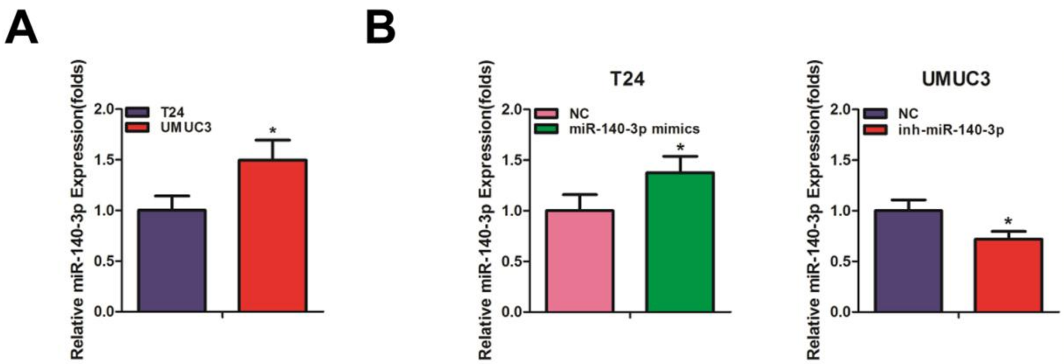

Supplementary Figure 1. (**A**) The levels of *miR-140-3p* in T24 and UMUC3 cells were compared using qRT-PCR. (**B**) *miR-140-3p* overexpression and knockdown in T24 and UMUC3 cells were verified using qRT-PCR. *$P < 0.05$ compared to controls.
